# Author Correction: Avalanches and edge-of-chaos learning in neuromorphic nanowire networks

**DOI:** 10.1038/s41467-024-54027-1

**Published:** 2024-11-11

**Authors:** Joel Hochstetter, Ruomin Zhu, Alon Loeffler, Adrian Diaz-Alvarez, Tomonobu Nakayama, Zdenka Kuncic

**Affiliations:** 1 School of Physics, University of Sydney, Sydney, NSW Australia; 2 International Center for Materials Nanoarchitectonics (WPI-MANA), National Institute for Materials Science (NIMS), Tsukuba, Ibaraki Japan; 3 Graduate School of Pure and Applied Sciences, University of Tsukuba, Tsukuba, Ibaraki Japan; 4 The University of Sydney Nano Institute, Sydney, NSW Australia

**Keywords:** Nanowires, Computational nanotechnology, Complex networks, Nonlinear phenomena, Phase transitions and critical phenomena

Correction to: *Nature Communications* 10.1038/s41467-021-24260-z, published online 29 June 2021

In this article the following equation in the methods was incorrectly given as:

$$=\frac{3{(2{m}_{\ast })}^{1/2}{e}^{5/2}{(\phi /e)}^{1/2}}{A2{h}^{2}{s}^{2}}\exp \left\{-\frac{4\pi {(2{m}_{\ast }e)}^{1/2}}{h}s{\left(\frac{\phi }{e}\right)}^{1/2}\right\}$$

And should have been:

$$=A\cdot \frac{3{(2{m}_{\ast })}^{1/2}{e}^{5/2}{(\phi /e)}^{1/2}}{2{h}^{2}s}\cdot \exp \left[-\frac{4\pi {(2{m}_{\ast }e)}^{1/2}}{h}s{\left(\frac{\phi }{e}\right)}^{1/2}\right].$$

Second, in this article figure 1b has an error in the circuit diagram. The original figure is:

and should be:

The original article has been corrected.

