# Supplementary material for: Avalanches and edge-of-chaos learning in neuromorphic nanowire networks
Source: Nat Commun. 2021 Jun 29;12:4008. doi: 10.1038/s41467-021-24260-z (PMC8242064; doi:10.1038/s41467-021-24260-z)
Supplement: Supplementary file 1 — Supplementary Information [file 41467_2021_24260_MOESM1_ESM.pdf]

Supplementary Information: Avalanches and edge-of-chaos learning in neuromorphic nanowire networks

Joel Hochstetter[1], Ruomin Zhu[1], Alon Loeffler[1], Adrian Diaz-Alvarez[2], Tomonobu Nakayama[1,2,3], and Zdenka Kuncic[1,2,4]

[1]School of Physics, University of Sydney, Sydney, NSW, 2006, Australia
[2]International Center for Materials Nanoarchitectonics (WPI-MANA), National Institute for Materials Science (NIMS), 1-1 Namiki, Tsukuba, Ibaraki, 305-0044, Japan
[3]Graduate School of Pure and Applied Sciences, University of Tsukuba, 1-1 Namiki, Tsukuba, Ibaraki, 305-0044, Japan
[4]The University of Sydney Nano Institute, Sydney, NSW, 2006, Australia

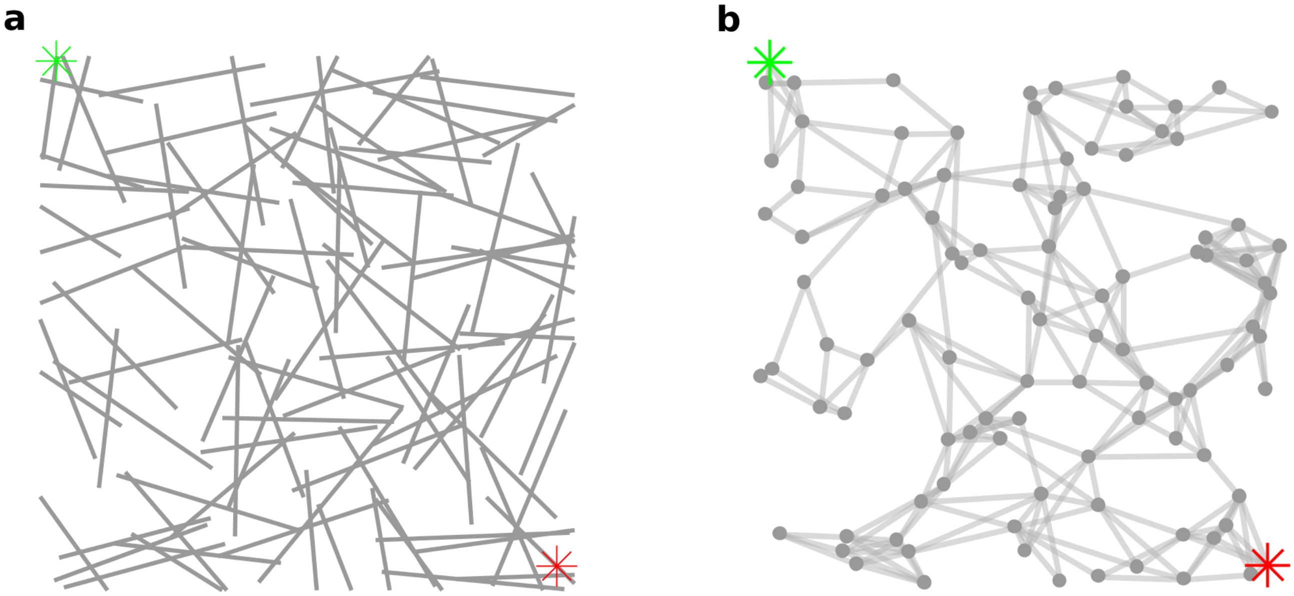

Supplementary Figure 1: Simulated self-assembled Ag nanowire networks. a Simulated network with 100 nanowires and 261 Ag|PVP|Ag junctions located at the intersection between nanowires. **b** Graphical representation of simulated network with nodes and edges corresponding to nanowires and junctions, respectively. Green and red asterisks refer to the source and drain, respectively.

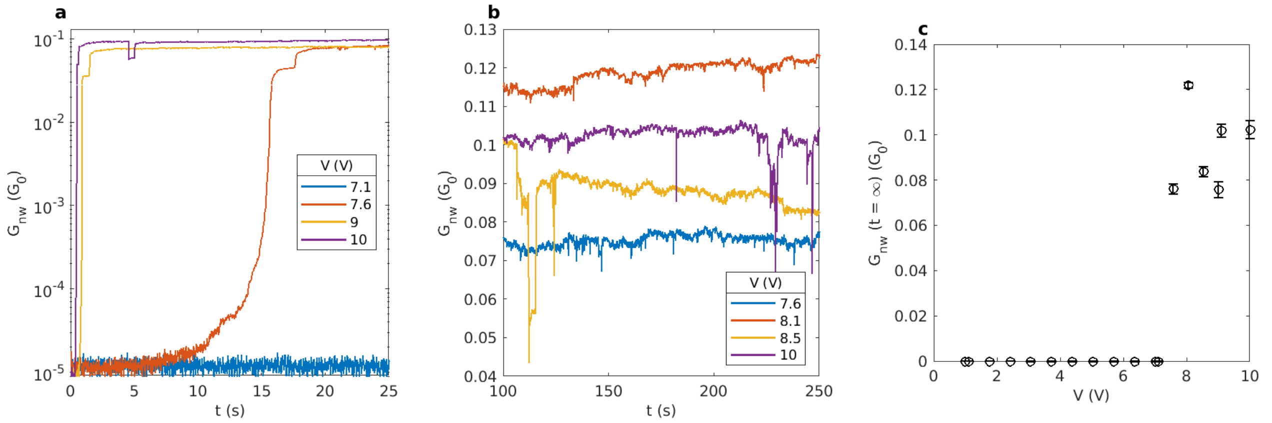

Supplementary Figure 2: Experimental observation of first order transition. **a** Experimental network conductance (G_{nw}) time-series at different voltages for networks initialised in low conductance state (LCS). **b** Persistent conductance fluctuations leads to transitions between multi-stable states. **c** Steady state conductance vs. voltage for experimental networks initialised in LCS. Networks were left with no applied voltage for 3 hours between measurements to allow junction filaments to reset. For points with $G_{\mathrm{nw}}(t = \infty) = 0$, current lies below experimental noise floor. Values and error bars correspond to mean and standard deviation of G_{nw} from $t = 210\,\mathrm{s}$ to $300\,\mathrm{s}$ after stimulus is applied. Details of experimental set-up are described in Methods.

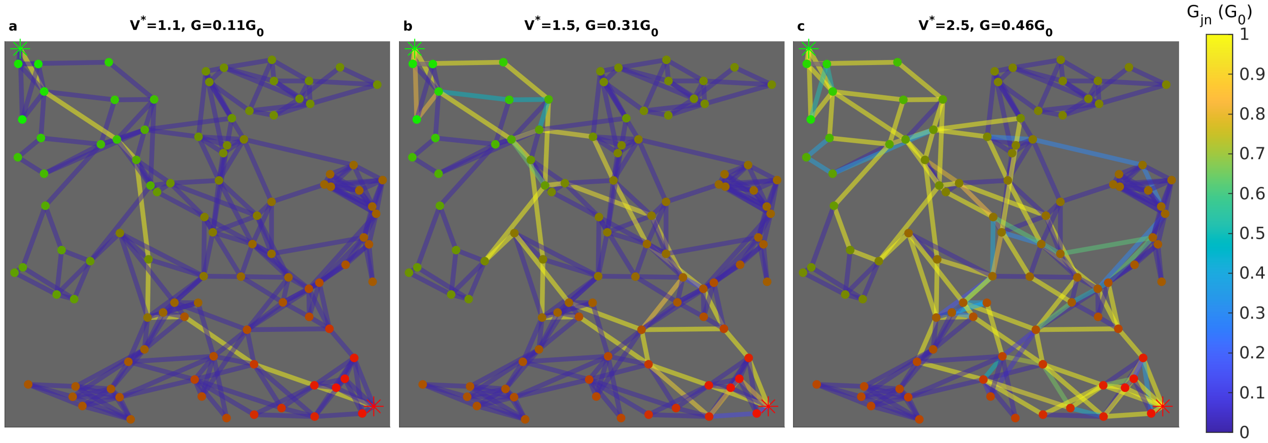

Supplementary Figure 3: Formation of parallel transport pathways. Snapshots of simulated junction conductance states (G_{jn}) for steady-state network for different applied voltages (**a**: $V^* = 1.1$, **b**: $V^* = 1.5$, **c**: $V^* = 2.5$, with $V^* = V/V_{\mathrm{th}}$), demonstrating the formation of multiple parallel transport pathways at higher voltages. A 100 nanowire, 261 junction network is used.

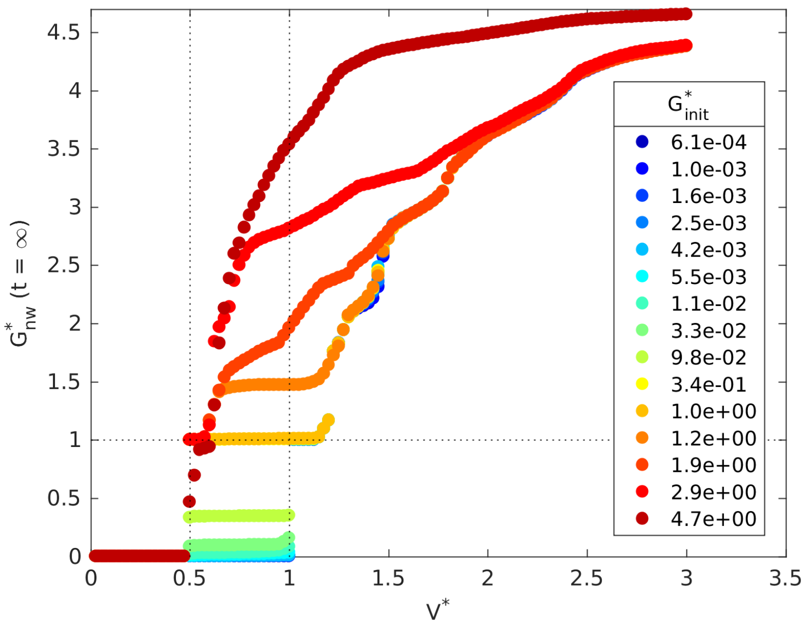

Supplementary Figure 4: Multi-stability under constant bias. For simulated networks with a wide range of initial states (taken by extracting filament states of the network at different times during a 1.8 V DC activation), the steady-state conductance ($G^*_{\mathrm{nw}}(t = \infty)$) is compared with applied voltage bias. Depending on the voltage networks of different initial states may converge to the same, or different, multi-stable steady states. G^*_{init} refers to network conductance of initial state. A 100 nanowire, 261 junction network is used.

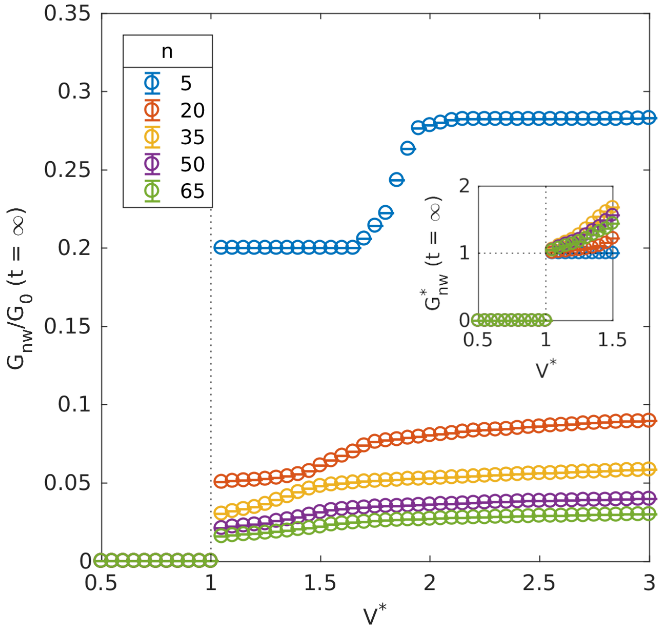

Supplementary Figure 5: $G - V$ transition for different electrode locations. Simulated steady-state conductance vs. voltage ($V^* = V/V_{\rm th}$) plots for different source-drain path lengths (n) on a network with 500 nodes and 1079 junctions. Networks are initialised to $\Lambda = 0$ for all junctions and the location of the first-order transition universally occurs at $V^* = 1$, with the network transitioning between $G^*_{\rm nw} = 0$ and $G^*_{\rm nw} = 1$.

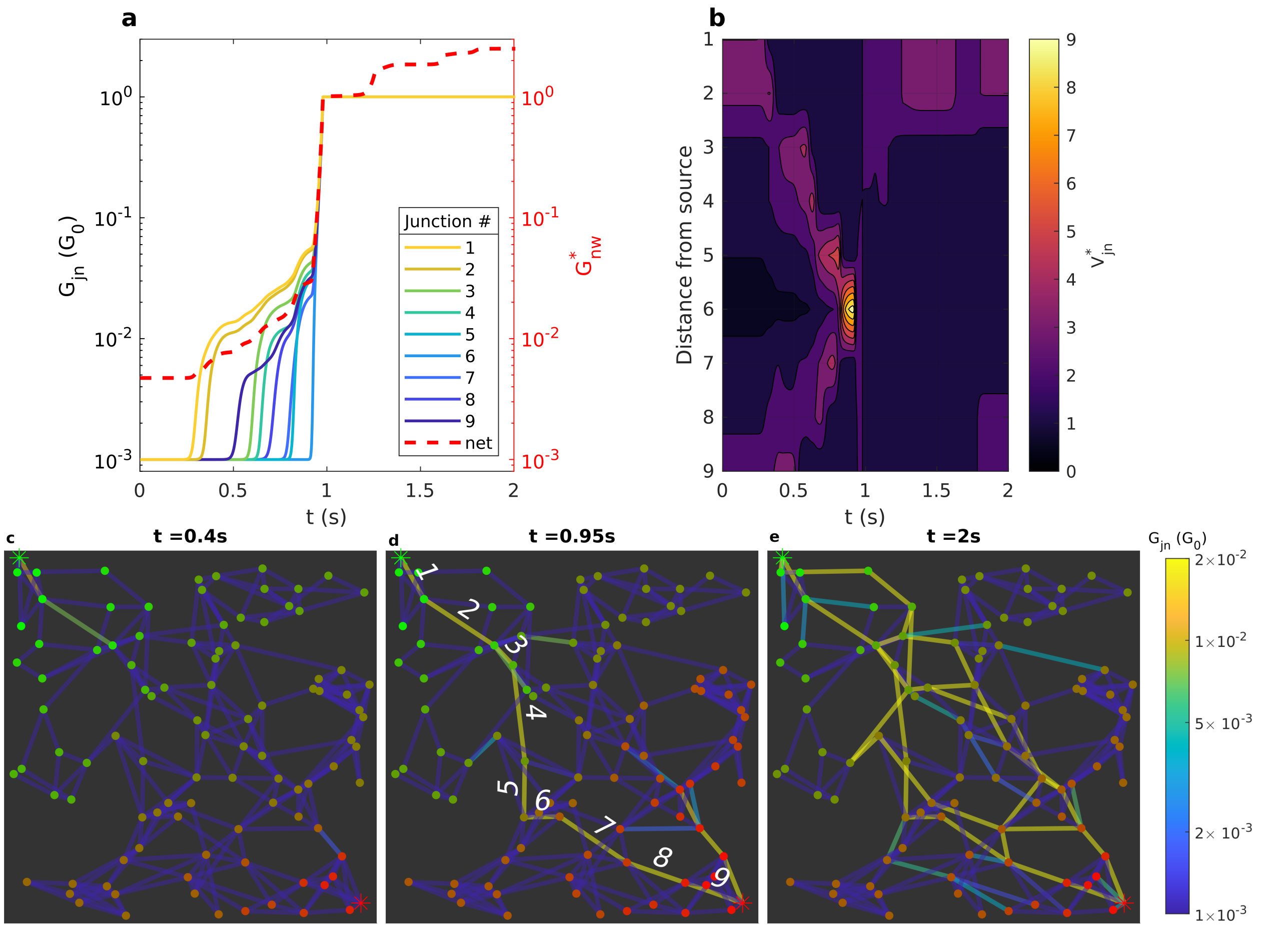

Supplementary Figure 6: Collective switching dynamics in a high voltage regime. **a** For voltage $V^* = 2 = 0.18\,\mathrm{V}$, simulated conductance states (in units of conductance quantum G_0) of each junction along shortest source-drain path. Junctions are numbered sequentially according to distance from source (#1 closest, #9 farthest). Network conductance, $G^*_{\mathrm{nw}}(t)$ (normalised by source-drain path length), is also shown. **b** Corresponding junction voltage time series. **c-e** Snapshots of junction conductance (edges shown on a logarithmic colourbar scale) at different time-points during activation. Node colour indicates voltage on nanowires between source (green asterisk) and drain (red asterisk). A 100 nanowire, 261 junction network is used.

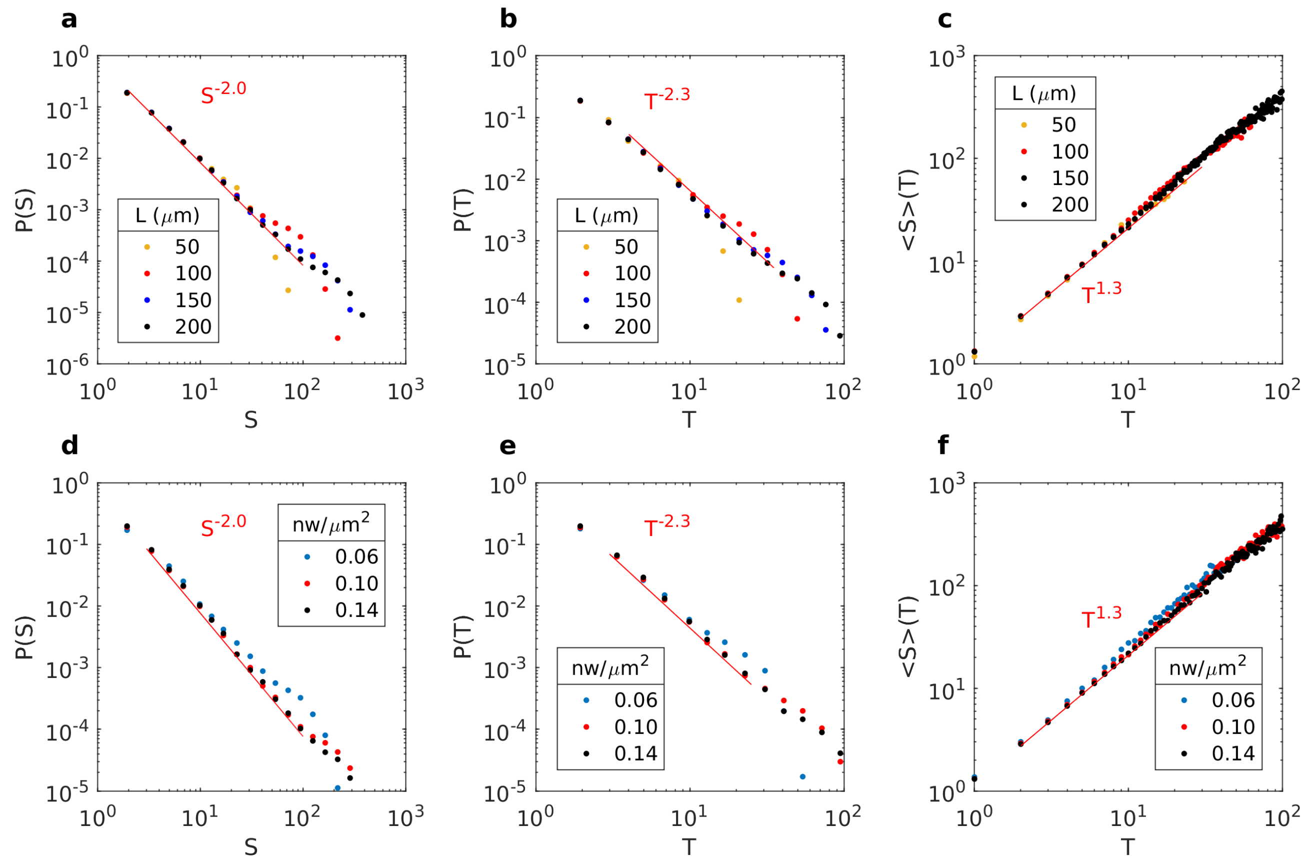

Supplementary Figure 7: Network dependence of avalanche statistics in simulation. Top row shows avalanche statistics for ensemble of 1000 NWNs at $V^* = 1$ with fixed density $0.10\,\mathrm{nw}(\mu\mathrm{m})^{-2}$, for increasing network size $L \times L$ where $L = 50, 100, 150, 200\,\mu$m. Bottom row shows avalanche statistics for ensemble of 1000 NWNs with fixed size $200 \times 200\,\mu\mathrm{m}^2$, for increasing density $0.06, 0.10, 0.14$ nw$(\mu$m$)^{-2}$. **a**, **d** show avalanche size distributions. **b**, **e** show avalanche life-time distributions. **c**, **f** show avalanche average size as function of life-time. Increasing the network density or size does not significantly affect the slope of the power law region, but increases the value of the power law break for $P(S)$, $P(T)$ and $\langle S\rangle(T)$. Logarithmic binning is used for histograms. The percolation threshold (minimum density such that the network has a large connected component) is ≈ 0.057 nw$(\mu$m$)^{-2}$ [1]. Avalanches are observed at densities well above the percolation threshold, unlike in nanoparticle networks [2].

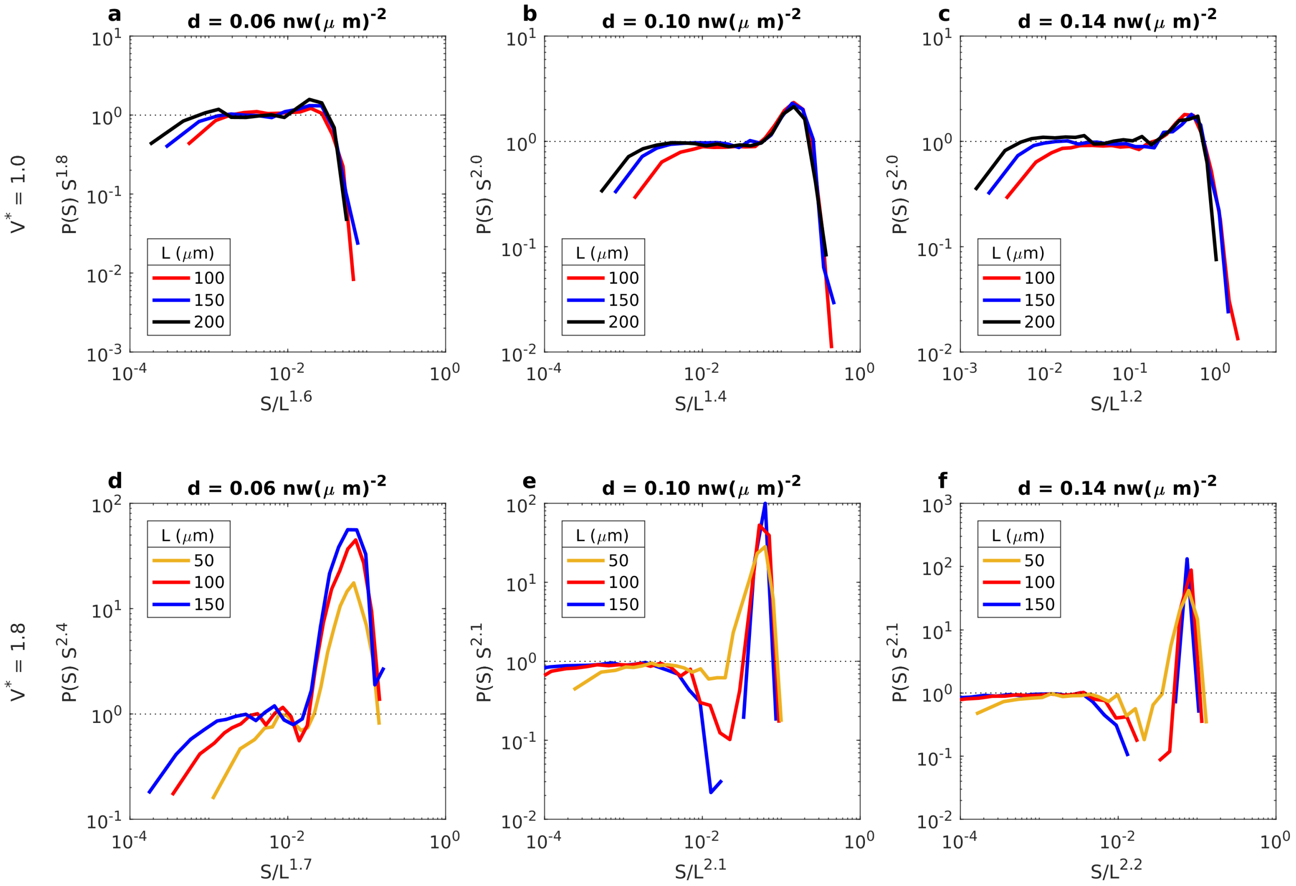

Supplementary Figure 8: Finite-size scaling analysis of avalanche size distributions in simulation. Re-scaled avalanche size distribution ($P(S)S^{\tau}$, for avalanche size S and exponent τ) is plotted against avalanche size re-scaled by network size (S/L^D, where D is another exponent called the avalanche dimension) for different network sizes ($L \times L$). Each column corresponds to network density (0.06, 0.10, 0.14 nw$(\mu\text{m})^{-2}$). The top row (**a-c**) and bottom row (**d-f**) show the corresponding plots for $V^* = 1$ and $V^* = 1.8$, respectively. The finite-size scaling (FSS) hypothesis states that at criticality, the re-scaled avalanche size distribution collapses onto a universal scaling function $G(x)$, where $G(x) =$ constant for $x \ll 1$ and $G(x) = 0$ for $x > 1$ [3, 4]. The collapse follows $P(S) = a\,S^{-\tau}G(S/s_c(L))$ where $s_c(L) = bL^D$, where a and b are constant metric factors. FSS breaks down at small avalanche sizes (below a lower cut-off), hence the deviation between collapsed curves for small S/L^D. The scaling collapse is performed manually to determine estimates of τ and D to the nearest 0.1. The method follows section 7.4 of Pruessner [4]. Briefly, $P(S)$ is logarithmically binned, τ is determined first such that the power-law part is horizontal, then D is chosen such that the peaks of the bumps at the right of the distribution have the same x-value. The height of the collapsed curves is multiplied by a constant factor, such that the power-law (flat) region has a y-value of 1. At $V^* = 1$, a collapse at each density is found, providing strong evidence that for *each* density at the critical voltage, NWNs exhibit avalanche criticality. The exponents found, $\tau = 1.8 - 2$ and $D = 1.2 - 1.6$, are consistent with the estimated avalanche exponents at $V^* = 1$ obtained by maximum likelihood fitting ($\tau \approx 1.95$). For $V^* = 1.8$, at each density for increasing network size the probability density (height of bump) grows, suggesting divergence in the $L \to \infty$ limit and indicating that NWNs at this voltage are super-critical.

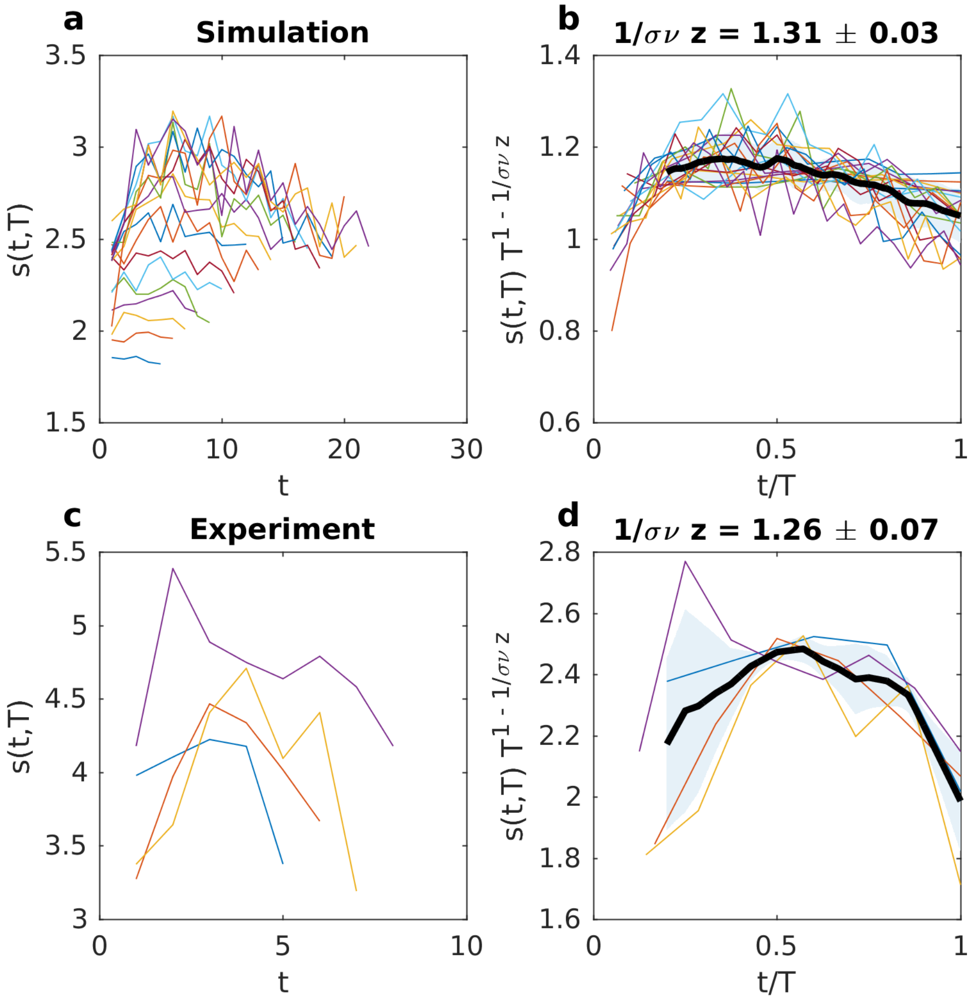

Supplementary Figure 9: Avalanche shape collapse analysis. Simulated **a** and experimental **c** data showing avalanche size ($s(t,T)$) as a function of time t for different lifetimes (T). Re-scaled size ($s(t,T)T^{1-1/\sigma\nu z}$) as a function of re-scaled time t/T are plotted for simulation **b** and experiment **d** respectively. Avalanches of different life-times (each curve) approximately converge onto a universal scaling function, as predicted by the theory of critical phenomena [5]. Each curve corresponds the average over avalanches of a certain lifetime. Only avalanches of lifetime $5 < T < x_{\mathrm{max}}$ (where $x_{\mathrm{max}} \approx 20$ is the cut-off of corresponding $P(T)$ distribution) and greater than 50 unique avalanches used. Exponent obtained using method from Marshall et. al. [6]. All trajectories for each avalanche duration are averaged to obtain characteristic shape. Re-scaled curves are linearly interpolated between $t/T = 0.2$ and $t/T = 1$ in increments between 0.01. At each interpolated points the mean (black curve) and standard deviation (light blue shading) are determined. $1/\sigma\nu z$ is determined to minimise mean square error divided by the square of the span of the re-scaled curves. The span is obtained by subtracting the minimum from the maximum value of $s(t,T)T^{1-1/\sigma\nu z}$ for a given $1/\sigma\nu z$. Error bars (1σ) are obtained by boostrapping with 1000 samples. An ensemble of 3000 networks of size $100 \times 100\,\mu\mathrm{m}^2$ and density $0.1\,\mathrm{nw}(\mu\mathrm{m})^{-2}$ at $V^* = 1$ are used for simulations. For experiment, datasets from 2 networks are combined to obtain sufficiently many avalanche statistics. Collapse can be improved by sampling a much larger number of avalanches.

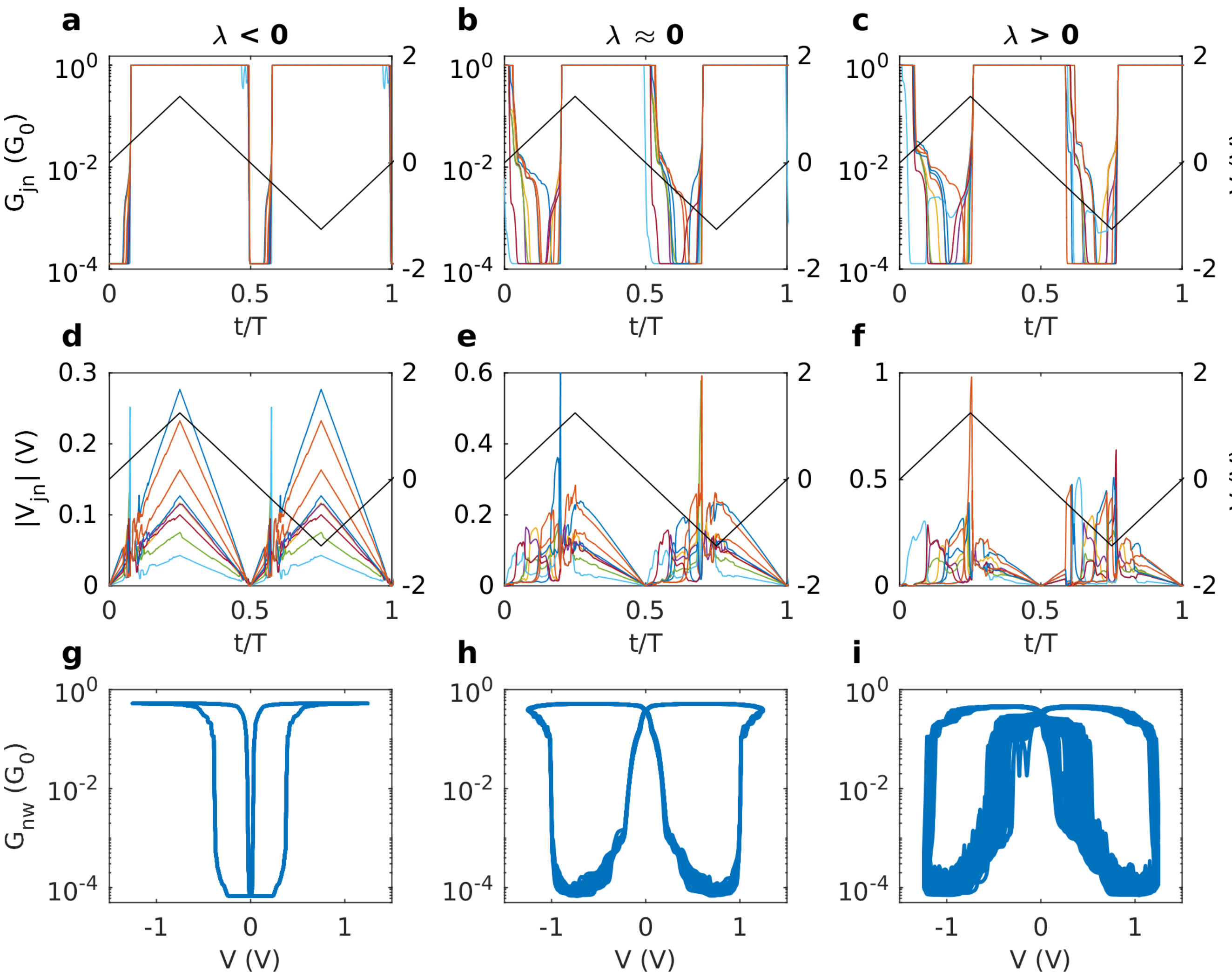

Supplementary Figure 10: Ordered ($\lambda < 0$), edge-of-chaos ($\lambda \approx 0$) and chaotic ($\lambda > 0$) junction states. Each column corresponds to a single network (100 nanowire, 261 junction) simulation for triangular AC input with $A = 1.25\,\mathrm{V}$ and $f = 0.1, 0.5, 0.85\,\mathrm{Hz}$ (left to right). First column (**a**, **d**): $f = 0.1\,\mathrm{Hz}$, $r = 7.7 \times 10^3$ and $\lambda = -2.6\,\mathrm{s}^{-1}$; all junction filament states return to 0 between cycles (reaching $V = 0$), resulting in symmetric repeatable $I - V$, $G_{\mathrm{nw}} - V$ and G_{jn} cycles. Second column (**b**, **e**) a state near the edge-of-chaos, with $f = 0.5\,\mathrm{Hz}$, $r = 7.1 \times 10^3$ and $\lambda = 0.4\,\mathrm{s}^{-1}$; network does not fully deactivate as polarity of voltage is reversed. Third column (**c**, **f**): $f = 0.85\,\mathrm{Hz}$, $r = 5.9 \times 10^3$ and $\lambda = 4.1\,\mathrm{s}^{-1}$; network trajectories are chaotic. First row: conductance of junctions G_{jn} along shortest path from source to drain, as a function of time t (in units of the period $T = 1/f$) for a single period, with triangular stimulus signal shown (black). Second row: corresponding magnitude of junction voltage, $|V_{\mathrm{jn}}|$. Third row: 20 cycles of a network $G - V$ curve plotted on a semilogarithmic scale. Corresponding network $I - V$ and $G - V$ curves on a linear scale are given in Fig. 7 in the main text.

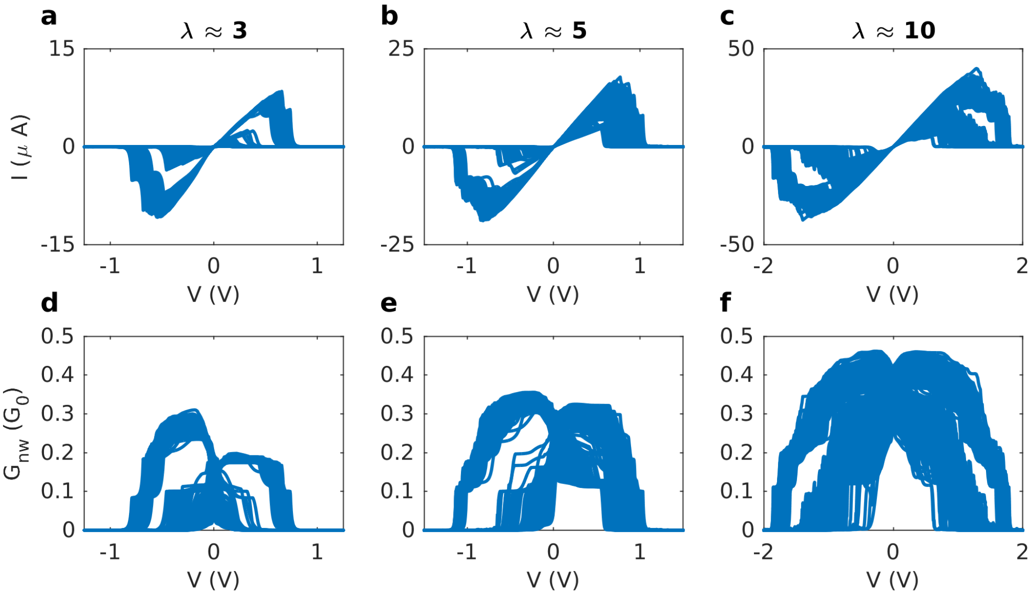

Supplementary Figure 11: Examples of chaotic trajectories with different Lyapunov exponents Each column corresponds to a single NWN simulation for a triangular AC input ($n = 20$) cycles, with $f = 1.5\,\text{Hz}$ and $A = 1.25, 1.5, 2\,\text{V}$ (left to right). Top row: $I - V$ curves, with arrows indicating direction of voltage control. Bottom row: corresponding network conductance, G_{nw}. First column (**a**, **d**): $V = 1.25\,\text{V}$, $r = 3.8 \times 10^3$ and $\lambda = 3.0\,\text{s}^{-1}$. Second column (**b**, **e**): $V = 1.5\,\text{V}$, $r = 4.1 \times 10^3$ and $\lambda = 5.0\,\text{s}^{-1}$. Third column (**c**, **f**): $V = 2\,\text{V}$, $r = 5.8 \times 10^3$ and $\lambda = 9.7\,\text{s}^{-1}$. A 100 nanowire, 261 junction network is used, but qualitatively similar results are found on a range of network sizes and densities. In these curves the network is inactive (low G_{nw}) at high V due to the lag between junction filament growth and pathway formation (paths of junctions with $G_{\text{jn}} = \text{G}_0$) across the network at high f.

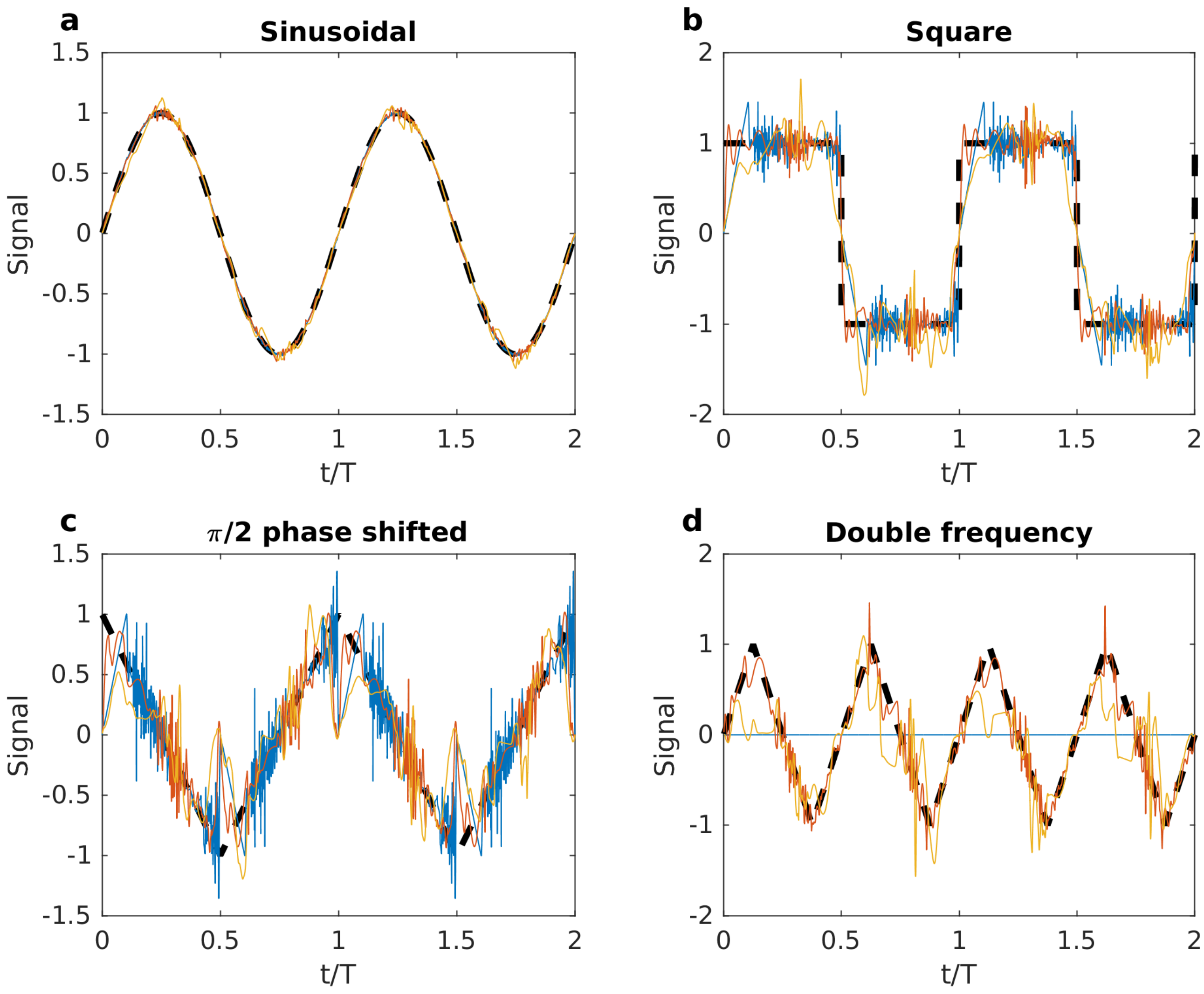

Supplementary Figure 12: Transformed signals from simulated non-linear transformation (NLT) task. Dotted black line shows target signal for each task (sin, square, phase shifted, double frequency). Each colour curve corresponds to a different input stimuli at amplitude of 1 V and Lyapunov exponent λ. Blue lines correspond to $f = 0.25\,\mathrm{Hz}$ and $\lambda = -1.46\,\mathrm{s}^{-1}$. The NLT accuracy of this ordered state is 99% for sine, 74% for square, 54% for phase shifted and 0% for double frequency. Red lines correspond to $f = 0.85\,\mathrm{Hz}$ and $\lambda = 0.02\,\mathrm{s}^{-1}$. The NLT accuracy of this edge-of-chaos state is 98% for sine, 81% for square, 56% for phase shifted and 75% for double frequency. Red lines correspond to $f = 1.00\,\mathrm{Hz}$ and $\lambda = 1.26\,\mathrm{s}^{-1}$. The NLT accuracy of this chaotic state is 93% for sine, 61% for square, 30% for phase shifted and 29% for double frequency. A network of 100 nodes, 261 junctions is used.

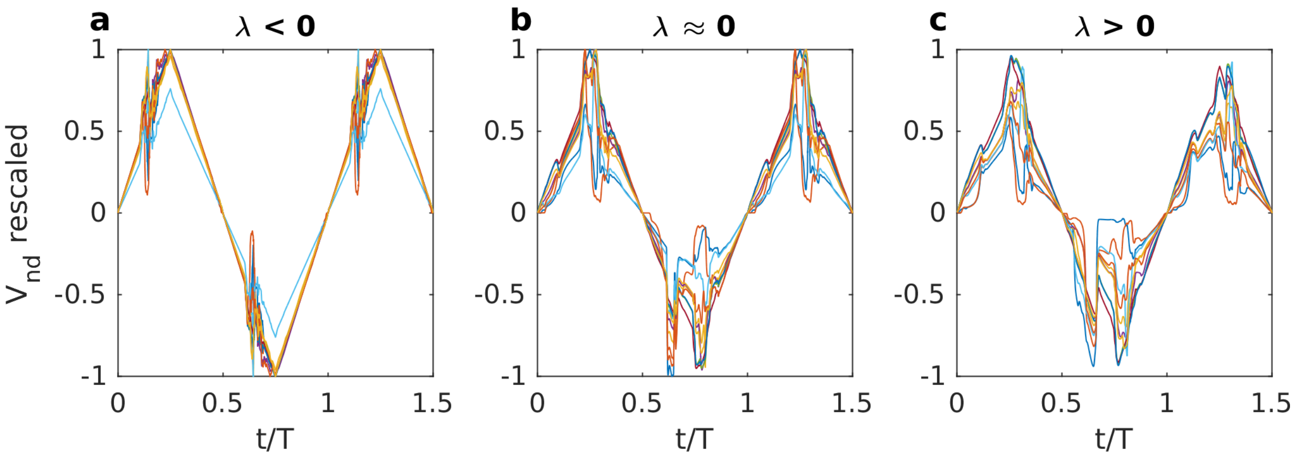

Supplementary Figure 13: Ordered ($\lambda < 0$), edge-of-chaos ($\lambda \approx 0$) and chaotic ($\lambda > 0$) node voltages for non-linear transformation simulations. Each plot shows the nanowire (node) voltages (V_{nd}) for the same 10 randomly chosen nodes for a 100 nanowire, 261 junction NWN under a triangular AC input with $A = 1\,\mathrm{V}$ and $f = 0.25, 0.85, 1.00\,\mathrm{Hz}$ (left to right). V_{nd} of each node is re-scaled so the maximum value is 1, curves are temporally rescaled by period $T = 1/f$. The corresponding Lyapunov exponents are $-1.45\,\mathrm{s}^{-1}$, $0.02\,\mathrm{s}^{-1}$ and $1.26\,\mathrm{s}^{-1}$. The corresponding performance in each task and transformed signals are shown in Supplementary Fig. 12.

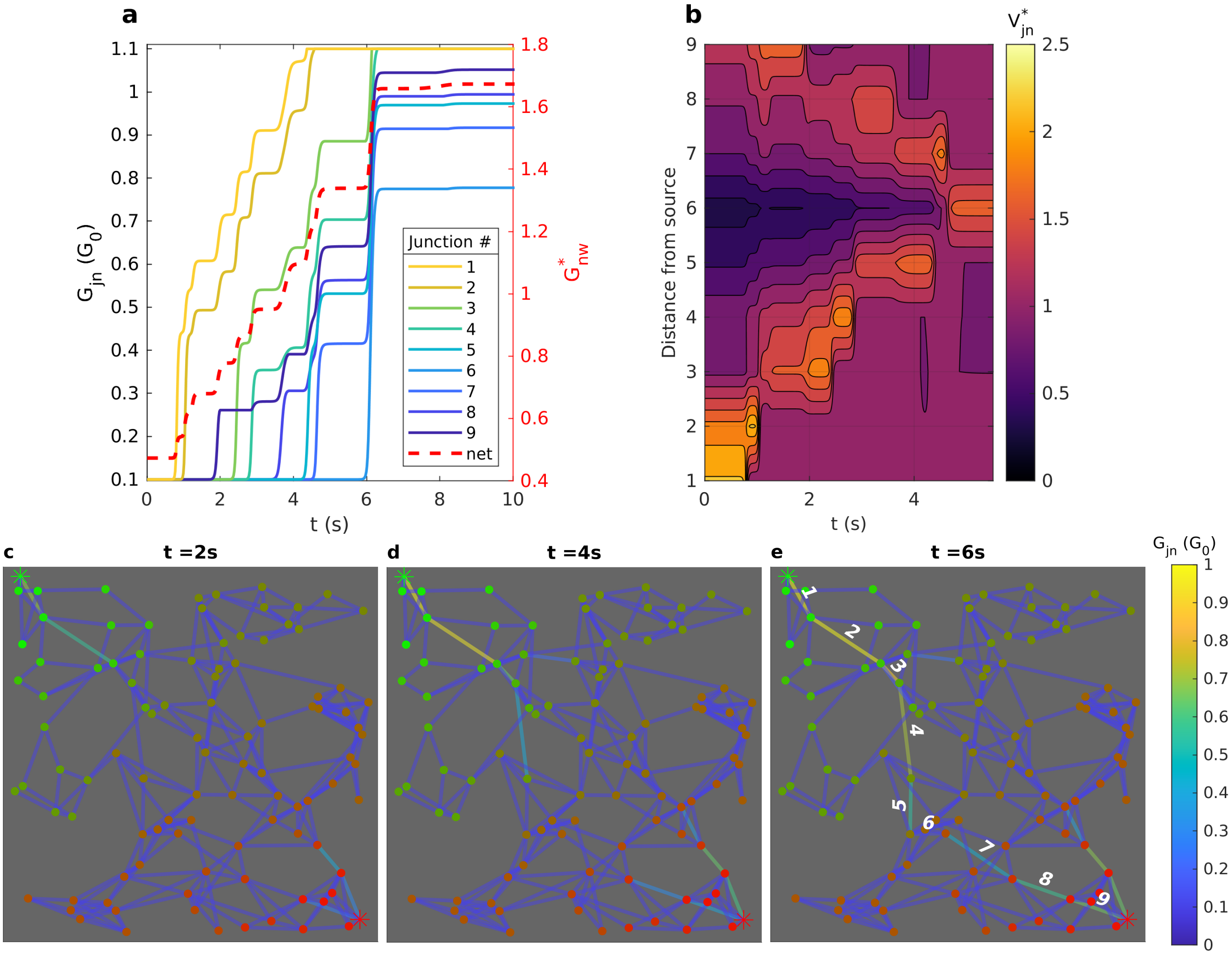

Supplementary Figure 14: Effect of $G_{\text{on}}/G_{\text{off}}$ on network dynamics under DC bias. Simulations using a 100 nanowire, 261 junction NWN with $G_{\text{on}}/G_{\text{off}} = 10$, compared with $G_{\text{on}}/G_{\text{off}} = 10^3$ in Fig. 3. **a** Conductance states of each junction, G_{jn}, along shortest source-drain path (in units of conductance quantum G_0). Junctions are numbered sequentially according to distance from source (#1 closest, #9 farthest). Network conductance, $G^*_{\text{nw}}(t)$ (normalised by source-drain path length), is also shown. **b** Corresponding junction voltage, $V^*_{\text{jn}} = V_{\text{jn}}/V_{\text{set}}$, dynamical re-distribution. **c-e** Snapshots of G_{jn} distribution at different time-points during network activation. Node colour indicates voltage on nanowires between source (green asterisk) and drain (red asterisk).

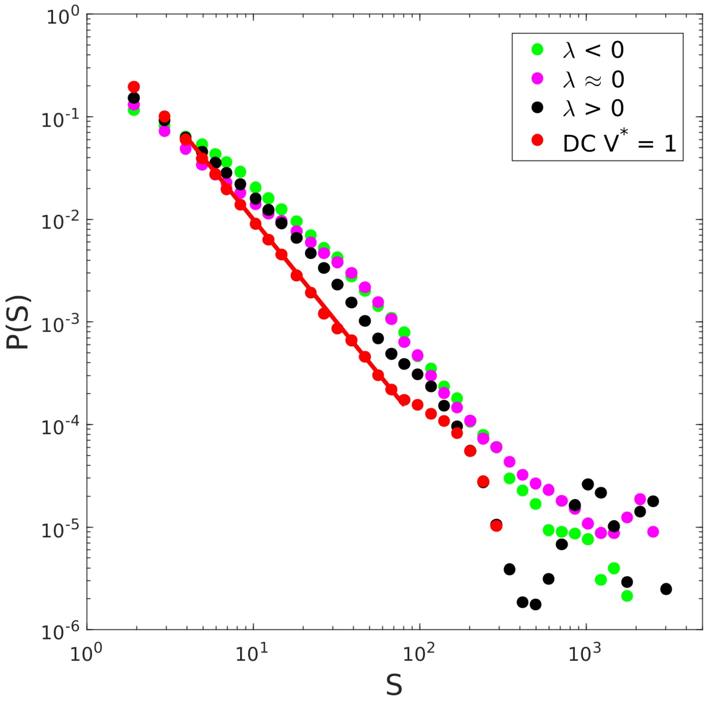

Supplementary Figure 15: AC avalanche dynamics. Characteristic AC avalanche size distributions. Statistics are produced from simulations on an ensemble 500 independently generated networks of size $150 \times 150\,\mu\text{m}^2$ with 2000 nanowires and 6000 junctions with pulses of amplitude $5\,\text{V}$ and frequencies $0.25\,\text{Hz}$ ($\lambda \approx -2\,\text{s}^{-1}$, green), $0.5\,\text{Hz}$ ($\lambda \approx 0\,\text{s}^{-1}$, pink), $1\,\text{Hz}$ ($\lambda \approx 2\,\text{s}^{-1}$, black). For comparison, red dots corresponds to $P(S)$ under DC at $V^* = 1$ for network of the same size (as in Supplementary Fig. 7). The corresponding maximum likelihood power-law fit as a red line indicating avalanche criticality in the DC case. However, any attempts to fit a power-law to green / pink / black curves fails Kolmogorov-Smirnov test ($p < 0.2$) unless range of fit is made very small ($x_{max}/x_{min} \lessapprox 2$), indicating deviation from avalanche criticality. Data is binned logarithmically.

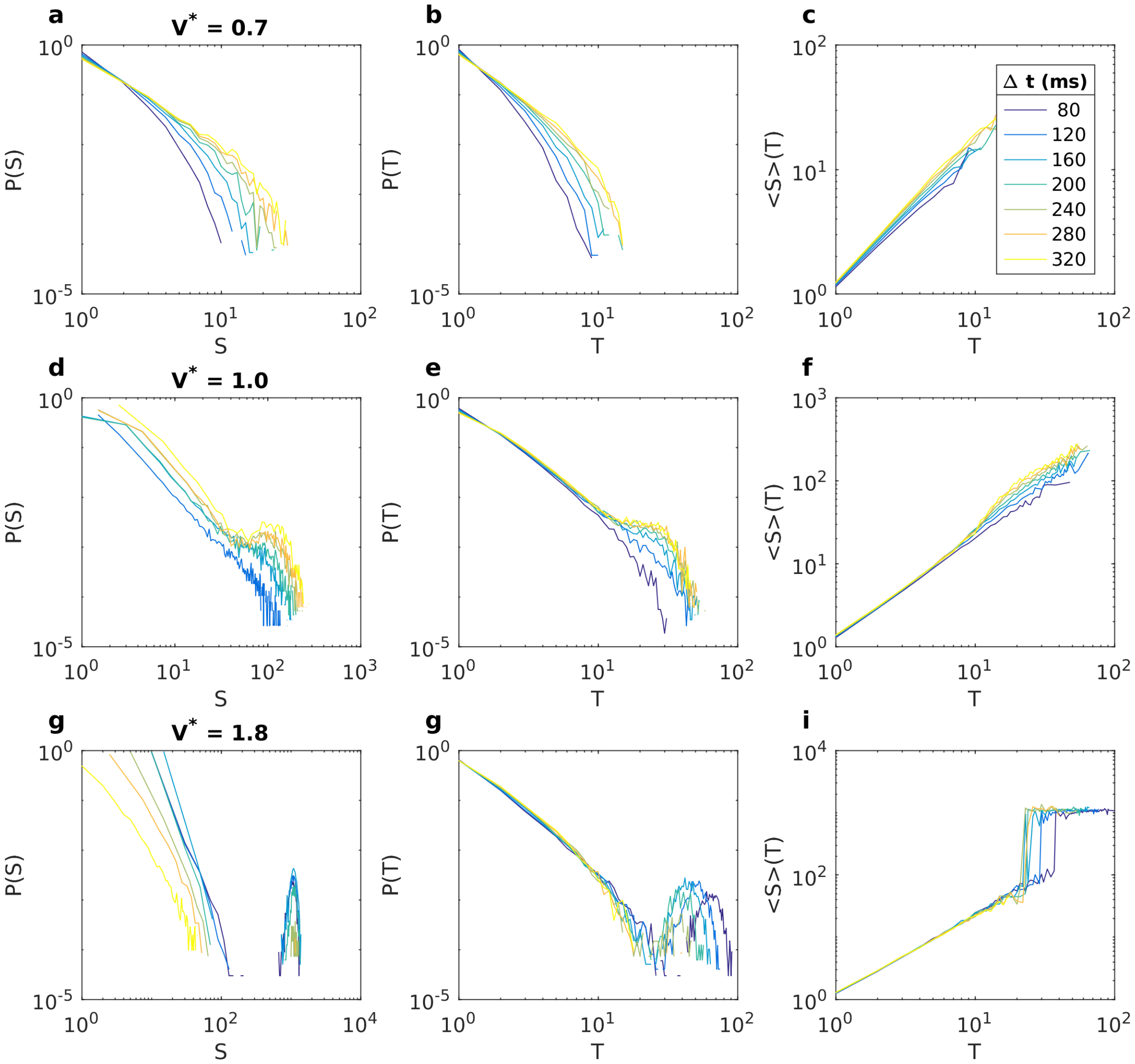

Supplementary Figure 16: Effect of changing frame on avalanche statistics for simulations. Each row shows effect of changing avalanche frame (Δt) on avalanche statistics at different voltages (top: $V^* = 0.7$, middle: $V^* = 1.0$, bottom: $V^* = 1.8$). **a**, **d**, **g** show avalanche size distributions ($P(S)$). **b**, **e**, **h** show avalanche life-time distributions ($P(T)$). **c**, **f**, **i** show avalanche average size ($\langle S\rangle(T)$) as a function of life-time. $\Delta t = 160\,\text{ms}$ is the average inter-event-interval at $V^* = 1$. An ensemble of 1000 networks of size $100\mu\text{m}\times 100\mu\text{m}$ and density $0.10\,\text{nw}(\mu\text{m})^{-2}$ is used. For critical states ($V^* = 1$) changing the frame width does not significantly change the slope, or shape of the curves, except at the tail where finite size effects are present. For sub-critical state ($V^* < 1$) and super-critical state ($V^* > 1$) distributions are more significantly altered, but retain similar qualitative shape when Δt is varied.

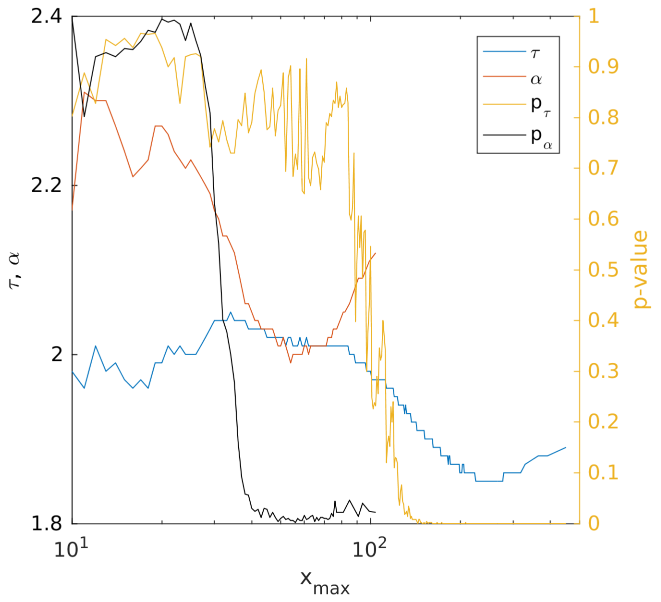

Supplementary Figure 17: Effect of cut-offs on fitted power-law exponents. Fitted exponents and p-value obtained by the Kolmolgorov-Smirnov test of avalanche distributions ($P(S)$, $P(T)$ with exponents τ, α respectively) for different upper cut-offs of fit (x_{max}) for simulations. Lower cut-offs (x_{min}) are fixed to 7 for both $P(S)$ and $P(T)$. An ensemble of 1000 networks of size 150μm$\times150\mu$m and density $0.10\,\mathrm{nw}(\mu\mathrm{m})^{-2}$ is used.

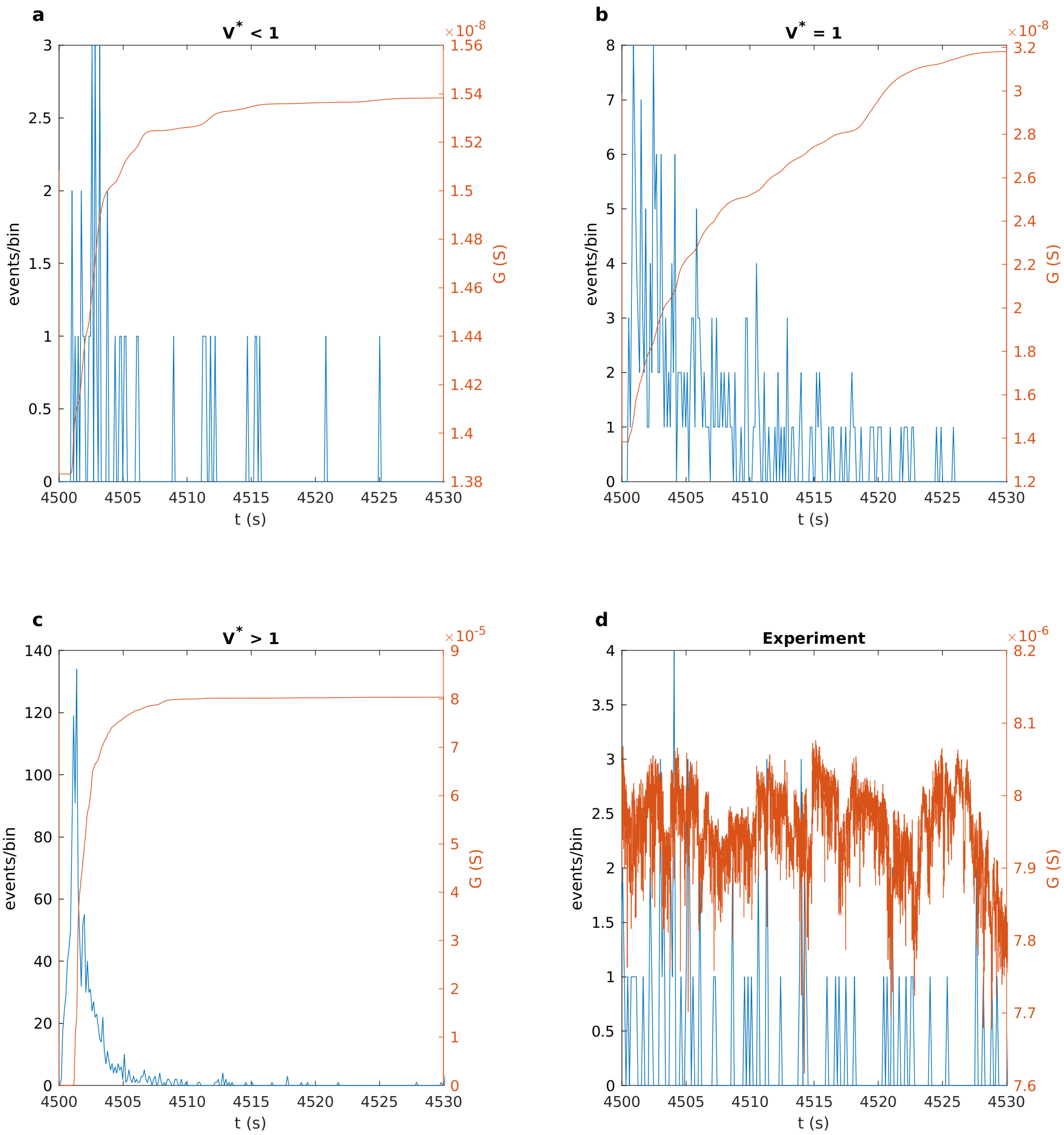

Supplementary Figure 18: Examples of avalanches in simulations and experiments. Example events per time bin (frame) and conductance time-series used in avalanche analysis. **(a-c)** correspond to data taken from one chosen network at $V^* = 0.7, 1.0, 1.8$ respectively. Avalanche distributions (averaging over 1000 independent networks of density $0.10\,\mathrm{nw}(\mu\mathrm{m})^{-2}$ and size $100\mu\mathrm{m}\times100\mu\mathrm{m}$) are shown in Fig. 5. Simulated avalanches are initiated by manually perturbing the network and allowing it relax to steady state conductance. **(d)** Example of experimental data and subsequent extracted events. In experiment avalanches are triggered by noise / junction breakdown events. For simulation at each V^* **(a-c)** the time-frame ($\Delta t = 160\,\mathrm{ms}$) corresponding to average inter-event-interval ($\langle\mathrm{IEI}\rangle$) at $V^* = 1$ is chosen to bin switching events. For, $V^* = 0.7$ and $V^* = 1.8$ $\langle\mathrm{IEI}\rangle$ is $755\,\mathrm{ms}$ and $25\,\mathrm{ms}$ respectively. In experiment, the time-frame ($\Delta t = 168\,\mathrm{ms}$) was chosen to correspond to the experimental $\langle\mathrm{IEI}\rangle$ for the dataset displayed.

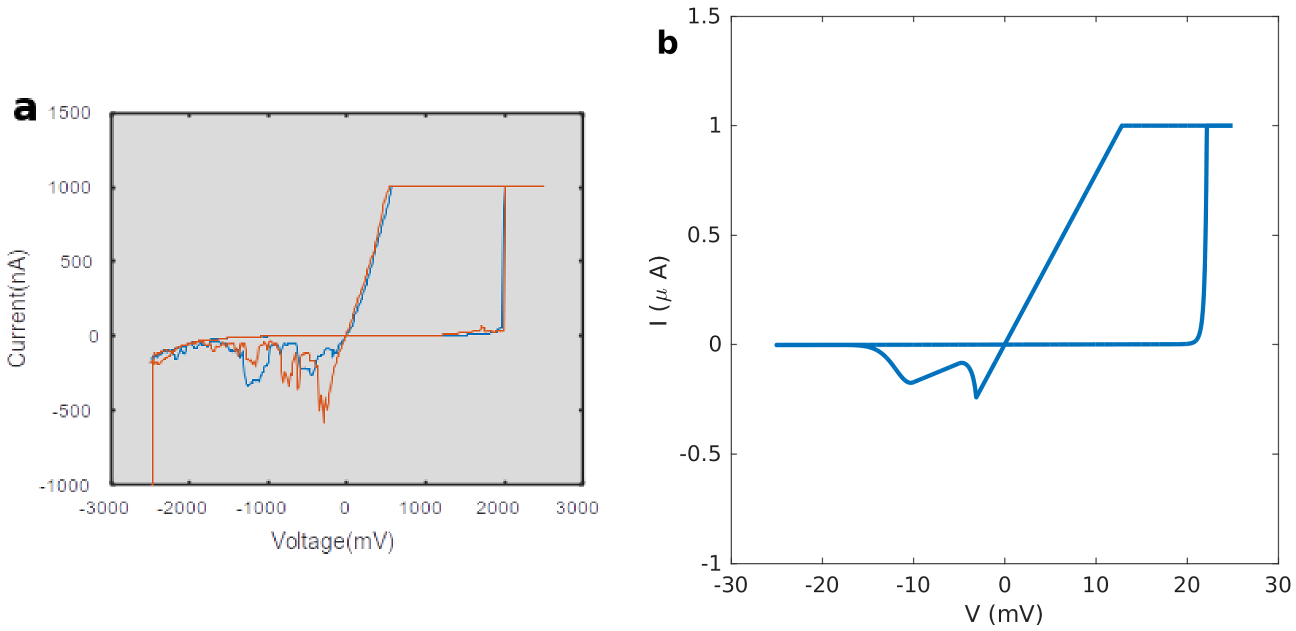

Supplementary Figure 19: Experimental $I - V$ curve for single Ag|PVP|Ag junction. **(a)** Experimental $I - V$ curve taken with $A = 2.5\,\mathrm{V}$ triangular wave and compliance current of $1\,\mu\mathrm{A}$. **(b)** Simulated $I - V$ curve under triangular wave with $A = 25\,\mathrm{mV}$, $f = 0.2\,\mathrm{Hz}$ and $b = 2$. A compliance current of $1\,\mu\mathrm{A}$ and initial filament state of $\Lambda = 7.5 \times 10^{-3}\,\mathrm{Vs}$ is used.

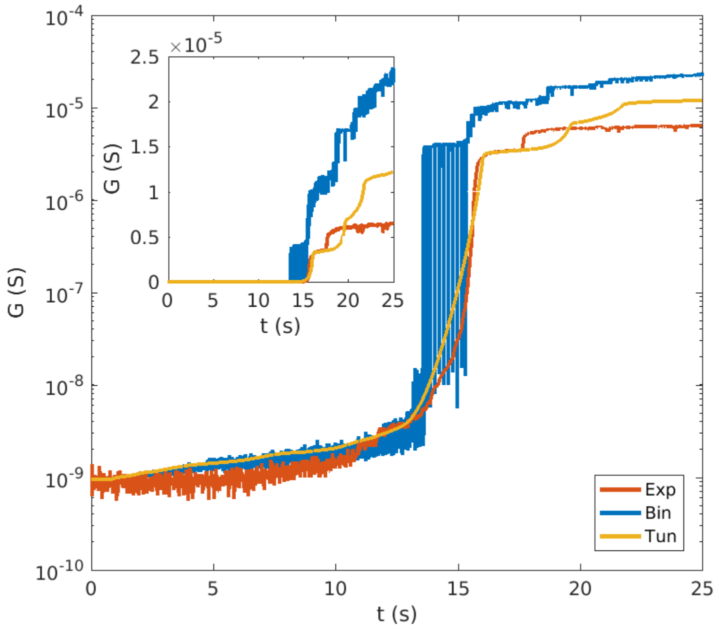

Supplementary Figure 20: Comparison between binary and tunnelling models. Network conductance time-series for binary and tunnelling models compared with experimental data. Binary model [7] has $G(\Lambda) = G_{\text{on}} = G_0$ if $|\Lambda| > \Lambda_{\text{crit}}$ and $G(\Lambda) = G_{\text{off}}$ otherwise. In simulation, a $100 \times 100\,\mu\text{m}^2$ network of density $0.1\,\text{nw}/\mu\text{m}^2$ with 992 nodes and 3008 junctions is used stimulated with a voltage of $V = 0.21\,\text{V}$ ($V^* \approx 1.1$). To achieve similar activation time and conductance as experiment $G_{\text{on}} = 5 \times 10^{-10}\,\text{S}$, $G_{\text{off}} = 7 \times 10^{-10}\,\text{S}$, $\Lambda_{\text{crit}} = 0.03\,\text{Vs}$ and $\Lambda_{\text{max}} = 0.045\,\text{Vs}$. Details of experimental set-up are provided in Methods, at a voltage of $V = 7.5\,\text{V}$ ($V^* \approx 1.1$). Note, the binary model is numerically unstable at low voltages ($V^* \sim 1$) as junctions can exhibit persistent switching between states across adjacent time-points (thick blue sections).

Supplementary Notes

Model validation

Model justification

According to the literature, resistive switching across Ag|PVP|Ag junctions is most likely mediated by conductive filament formation (and dissolution) due to electrochemical metallisation [8]. This has been studied in Ag-PVP-Ag nanowire-nanowire junctions [9–11] and in PVP coated Ag nanoparticle films above the percolation threshold [12]. Furthermore, Ag filament formation across Ag|PVP|Ag junctions has been directly visualised by SEM [13].

The modelling approach chosen here uses a simple 'memristive' model [14–16] that captures the basic phenomenology of voltage-controlled junction switching observed experimentally (Supplementary Fig. 19). A junction's conductance $G_{\mathrm{jn}} = I/V$ varies between two states (G_{off} and G_{on}) separated by several orders of magnitude. When junction voltage exceeds a set threshold V_{set}, a junction switches between these states in a non-linear manner. Upon reversal of bias, the junction reverts to a low conductance state when the voltage is sufficiently high. The junction can also decay to a low conductance state when the voltage (polarity independent) drops below V_{reset} [17]. Resistive switching is repeatable on adjacent cycles after an initial transient period.

While not as comprehensive as the model proposed in e.g. Ref. [18], this model improves on previous models of the dynamics of Ag|PVP|Ag nanowire junctions [7, 17] by including tunnelling [19] which is important at low voltage regimes (Supplementary Fig. 20) due to the nanoscale dimensions of junctions.

Parameter dependence

The qualitative behaviour of the simulations does not have a strong dependence on model parameters:

- V_{set} and V_{reset} affect the voltages at which individual junctions, and hence the network as a whole, activates or deactivates, respectively. When the ratio $V_{\mathrm{set}}/V_{\mathrm{reset}}$ remains fixed, identical behaviour is obtained by re-scaling the voltage applied to networks, and re-scaling time.
- Λ_{crit} sets the time-scale for filament formation. Λ_{max} denotes the amount of memory of the junction to its high conductance state; increasing Λ_{max} increases junction memory and hence time-scale of their deactivation when voltage is reduced below V_{reset}. b scales the rate of filament decay relative to growth.
- ϕ sets the tunnelling barrier height. As ϕ increases, G_{jn} approaches a step-function in Λ. As ϕ reduces, the exponential decay of filaments changes over a longer distance. A ϕ chosen based off material properties better qualitatively reproduces experimental data than a step function (Supplementary Fig. 20). The range of tunnelling (determined by ϕ) does not affect the observation of avalanches or chaotic dynamics. Further, it is not necessary to remain in the tunnelling regime; all junctions which activate/deactivate will pass through this regime.
- The effect of $G_{\mathrm{on}}/G_{\mathrm{off}}$ is described in Discussion and is compared in Supplementary Fig. 14 and Fig. 3.
- For the order-chaos transition results: Changing b, the ratios $\Lambda_{\mathrm{max}}/\Lambda_{\mathrm{crit}}$ and $V_{\mathrm{set}}/V_{\mathrm{reset}}$ affect the frequencies and voltages at which chaotic, edge-of-chaos and ordered dynamics are reached. However, for each set of model parameters we explored the network can be tuned into these three regimes by sweeping frequency and amplitude of the driving signal. The same qualitative trend from Fig. 8 holds: above a certain amplitude increasing frequency causes Lyapunov exponent to first increase (from below to above $\lambda = 0$) then decrease (falling below $\lambda = 0$, for sufficiently high f). The observation of chaos in this model requires a combination between polarity-driven and decay-driven off-switching for junctions.

Model assumptions

- Junctions are assumed to be deterministic. To study the effects of junction noise on network dynamics, a noise term could be added to equation 2.
- The model assumes homogeneity of junction parameters between junctions. In reality variations in PVP thickness and contact geometry will mean V_{set}, V_{reset}, Λ_{crit}, Λ_{max} will differ from junction to junction. The model could be modified to draw each of these parameters from a probability distribution.
- Capacitive effects, which may play a role in low current (pA) regimes, are not considered here. This model could be modified by considering each junction as an RC circuit.
- Breakdown of junctions [20] or nanowires [11] due to electromigration is omitted. These effects may play a role for modelling persistent fluctuations of networks on long time-scales (Supplementary Fig. 5b). A model similar to used for filament breakdown between nanoparticles [21] could be used.

Supplementary References

1. Li, J. & Zhang, S.-L. Finite-size scaling in stick percolation. *Physical Review E* **80,** 040104 (2009).
2. Mallinson, J. B. *et al.* Avalanches and criticality in self-organized nanoscale networks. *Science Advances* **5,** eaaw8438 (2019).
3. Cardy, J. *Scaling and Renormalization in Statistical Physics* (Cambridge University Press, 1996).
4. Pruessner, G. *Self-Organised Criticality* (Cambridge University Press, Cambridge, 2012).
5. Sethna, J. P., Dahmen, K. A. & Myers, C. R. Crackling noise. *Nature* **410,** 242–250 (2001).
6. Marshall, N. *et al.* Analysis of Power Laws, Shape Collapses, and Neural Complexity: New Techniques and MATLAB Support via the NCC Toolbox. *Frontiers in Physiology* **7,** 1–18 (2016).
7. Kuncic, Z. *et al.* Emergent brain-like complexity from nanowire atomic switch networks: Towards neuromorphic synthetic intelligence. *2018 IEEE 18th International Conference on Nanotechnology (IEEE-NANO),* https://doi.org/10.1109/NANO.2018.8626236 (2018).
8. Kuncic, Z. & Nakayama, T. Neuromorphic nanowire networks: principles, progress and future prospects for neuro-inspired information processing. *Advances in Physics: X* **6,** 1894234 (2021).
9. Bellew, A. T., Manning, H. G., Gomes da Rocha, C., Ferreira, M. S. & Boland, J. J. Resistance of Single Ag Nanowire Junctions and Their Role in the Conductivity of Nanowire Networks. *ACS Nano* **9,** 11422–11429 (2015).
10. Manning, H. G. *et al.* Emergence of winner-takes-all connectivity paths in random nanowire networks. *Nature Communications* **9,** 1–9 (2018).
11. Milano, G. *et al.* Brain-Inspired Structural Plasticity through Reweighting and Rewiring in Multi-Terminal Self-Organizing Memristive Nanowire Networks. *Advanced Intelligent Systems* **2,** 2000096 (2020).
12. Sandouk, E. J., Gimzewski, J. K. & Stieg, A. Z. Multistate resistive switching in silver nanoparticle films. *Science and Technology of Advanced Materials* **16,** 045004 (2015).
13. Yang, H. *et al.* Controlled Growth of Fine Multifilaments in Polymer-Based Memristive Devices Via the Conduction Control. *ACS Applied Materials & Interfaces* **12,** 34370–34377 (2020).
14. Strukov, D. B., Snider, G. S., Stewart, D. R. & Williams, R. S. The missing memristor found. *Nature* **453,** 80–83 (2008).
15. Kvatinsky, S., Ramadan, M., Friedman, E. G. & Kolodny, A. VTEAM: A General Model for Voltage-Controlled Memristors. *IEEE Transactions on Circuits and Systems II: Express Briefs* **62,** 786–790 (2015).
16. Caravelli, F. & Carbajal, J. Memristors for the Curious Outsiders. *Technologies* **6,** 118 (2018).
17. Diaz-Alvarez, A. *et al.* Emergent dynamics of neuromorphic nanowire networks. *Scientific Reports* **9,** 14920 (2019).
18. Menzel, S. Comprehensive modeling of electrochemical metallization memory cells. *Journal of Computational Electronics* **16,** 1017–1037 (2017).
19. Simmons, J. G. Generalized Formula for the Electric Tunnel Effect between Similar Electrodes Separated by a Thin Insulating Film. *Journal of Applied Physics* **34,** 1793–1803 (1963).
20. Song, T.-B. *et al.* Nanoscale Joule Heating and Electromigration Enhanced Ripening of Silver Nanowire Contacts. *ACS Nano* **8,** 2804–2811 (2014).
21. Pike, M. D. *et al.* Atomic Scale Dynamics Drive Brain-like Avalanches in Percolating Nanostructured Networks. *Nano Letters* **20,** 3935–3942 (2020).
